# Supplementary material for: Bovine tuberculosis breakdown duration in cattle herds: an investigation of herd, host, pathogen and wildlife risk factors
Source: PeerJ. 2020 Feb 3;8:e8319. doi: 10.7717/peerj.8319 (PMC7003687; doi:10.7717/peerj.8319)
Supplement: Table S1 [file peerj-08-8319-s002.docx]

**Supplementary Material, Table 1**: Results of the negative binomial count model of breakdown duration (untransformed model coefficients)

| Random Effects | **Variance** | **Std.Dev.** |  |  |
| --- | --- | --- | --- | --- |
| herd_id (Intercept) | 0.048 | 0.218 |  |  |
| dvo (Intercept) | 0.005 | 0.068 |  |  |
| year (Intercept) | 0.006 | 0.077 |  |  |
|  |  |  |  |  |
|  |  |  |  |  |
|  |  |  |  |  |
| Fixed effects | **Estimate** | **Std. Error** | **z value** | **p** |
| Intercept | 4.881 | 0.052 | 93.391 | <0.001 |
| log(herd_size) | 0.045 | 0.005 | 9.631 | <0.001 |
| log(outbreak_reactors) | 0.047 | 0.006 | 7.558 | <0.001 |
| log(mean_patch_prev) | 0.037 | 0.014 | 2.589 | 0.010 |
| log(MLVA_Richness) | 0.484 | 0.014 | 34.518 | <0.001 |
| LRS_binary1 | 0.113 | 0.011 | 9.865 | <0.001 |
| associated_herds_binary1 | 0.093 | 0.013 | 7.380 | <0.001 |
| previous_breakdown | 0.039 | 0.012 | 3.255 | 0.001 |
