# Supplementary material for: Bovine tuberculosis breakdown duration in cattle herds: an investigation of herd, host, pathogen and wildlife risk factors
Source: PeerJ. 2020 Feb 3;8:e8319. doi: 10.7717/peerj.8319 (PMC7003687; doi:10.7717/peerj.8319)
Supplement: Table S2 [file peerj-08-8319-s003.docx]

**Supplementary Material, Table 2**: Results of the negative binomial count model of breakdown duration, with *log* MLVA richness included with a quadratic term (untransformed model coefficients).

| Random effects | | **Variance** | **Std.Dev** |  |
| --- | --- | --- | --- | --- |
| herd_id |  | 0.048 | 0.218 |  |
| Dvo |  | 0.005 | 0.068 |  |
| Year |  | 0.006 | 0.077 |  |
|  |  |  |  |  |
|  |  |  |  |  |
|  | |  |  |  |
| Fixed effects | **Estimate** | **Std. Error** | **z value** | **p** |
| (Intercept) | 4.883 | 0.052 | 94.001 | <0.001 |
| log(herd_size) | 0.045 | 0.005 | 9.733 | <0.001 |
| log(outbreak_reactors) | 0.049 | 0.006 | 7.778 | <0.001 |
| log(mean_patch_prev) | 0.037 | 0.014 | 2.602 | 0.01 |
| log(MLVA_Richness) | 0.311 | 0.034 | 9.092 | <0.001 |
| LRS_binary1 | 0.111 | 0.011 | 9.680 | <0.001 |
| associated_herds_binary1 | 0.091 | 0.013 | 7.245 | <0.001 |
| previous_breakdown | 0.037 | 0.012 | 3.063 | <0.001 |
| I(log(MLVA_Richness)^2) | 0.158 | 0.029 | 5.520 | <0.001 |
