# Supplementary material for: Bovine tuberculosis breakdown duration in cattle herds: an investigation of herd, host, pathogen and wildlife risk factors
Source: PeerJ. 2020 Feb 3;8:e8319. doi: 10.7717/peerj.8319 (PMC7003687; doi:10.7717/peerj.8319)
Supplement: Table S3 [file peerj-08-8319-s004.docx]

**Supplementary Material, Table 3**: Results of the ordinal model of breakdown duration (untransformed model coefficients).

| Random effects | |  |  |  |
| --- | --- | --- | --- | --- |
| Groups |  | **Variance** | **Std.Dev.** |  |
| herd_id |  | 0.338 | 0.581 |  |
| dvo |  | 0.103 | 0.320 |  |
| year |  | 0.101 | 0.318 |  |
|  | | | | |
|  |  |  |  |  |
|  | |  |  |  |
| Fixed Effects | **Estimate** | **Std. Error** | **z value** | **p** |
| log(herd_size) | 0.230 | 0.024 | 9.549 | <0.001 |
| log(outbreak_reactors) | 0.295 | 0.033 | 9.019 | <0.001 |
| log(mean_patch_prev) | 0.179 | 0.071 | 2.517 | 0.012 |
| log(MLVA_Richness) | 1.954 | 0.079 | 24.690 | <0.001 |
| LRS_binary1 | 0.581 | 0.060 | 9.677 | <0.001 |
| associated_herds_binary1 | 0.401 | 0.062 | 6.526 | <0.001 |
