# Supplementary material for: Bovine tuberculosis breakdown duration in cattle herds: an investigation of herd, host, pathogen and wildlife risk factors
Source: PeerJ. 2020 Feb 3;8:e8319. doi: 10.7717/peerj.8319 (PMC7003687; doi:10.7717/peerj.8319)
Supplement: Table S4 [file peerj-08-8319-s005.docx]

**Supplementary Material, Table 4**: Results of (a) a negative binomial count model of breakdown duration and (b) a Gaussian GLM of breakdown duration (for illustrative purposes), with DVO and *log* main sett density as non-interacting predictors (untransformed model coefficients).

|  |  |  |  |  |  |  |  |  |
| --- | --- | --- | --- | --- | --- | --- | --- | --- |
|  | **Negative Binomial** | | | | **Gaussian GLM** | | | |
| **Coefficients:** | **Est** | **Std. Error** | **t** | **p** | **Est** | **Std. Error** | **t** | **p** |
| (Intercept) | 5.48 | 0.02 | 289. | < 2e-16 | 239.75 | 5.69 | 42.15 | < 2e-16 |
| dvoBallymena | -0.18 | 0.03 | -6.19 | 0.00 | -40.20 | 8.86 | -4.54 | 0.00 |
| dvoColeraine | -0.11 | 0.02 | -4.32 | 0.00 | -24.09 | 7.32 | -3.29 | 0.00 |
| dvoDungannon | 0.01 | 0.03 | 0.56 | 0.58 | 3.37 | 7.83 | 0.43 | 0.67 |
| dvoEnniskillen | -0.20 | 0.02 | -8.13 | 0.00 | -44.06 | 7.47 | -5.90 | 0.00 |
| dvoLarne | -0.05 | 0.03 | -1.71 | 0.09 | -12.59 | 9.28 | -1.36 | 0.17 |
| dvoLondonderry | -0.22 | 0.04 | -5.83 | 0.00 | -46.69 | 11.14 | -4.19 | 0.00 |
| dvoNewry | 0.11 | 0.02 | 4.73 | 0.00 | 27.41 | 6.82 | 4.02 | 0.00 |
| dvoNewtownards | -0.05 | 0.02 | -2.10 | 0.04 | -11.66 | 7.37 | -1.58 | 0.11 |
| dvoOmagh | -0.12 | 0.02 | -5.04 | 0.00 | -26.13 | 6.89 | -3.79 | 0.00 |
| log(main_sett) | 0.01 | 0.02 | 0.63 | 0.53 | 1.89 | 5.76 | 0.33 | 0.74 |
