# Supplementary material for: Bovine tuberculosis breakdown duration in cattle herds: an investigation of herd, host, pathogen and wildlife risk factors
Source: PeerJ. 2020 Feb 3;8:e8319. doi: 10.7717/peerj.8319 (PMC7003687; doi:10.7717/peerj.8319)
Supplement: Table S5 — The interaction between log main sett and DVO was significant (negative binomial: χ2 = 24.24, df = 9, p = 0.004); untransformed model coefficients. [file peerj-08-8319-s006.docx]

**Supplementary Material, Table 5**: Results of (a) a negative binomial count model of breakdown duration and (b) a Gaussian GLM (for illustrative purposes only) of breakdown duration, with DVO and *log* main sett density as interacting predictors. The interaction between *log* main sett and DVO was significant (negative binomial: χ² = 24.24, df = 9, *p* = 0.004); untransformed model coefficients.

|  | **Negative Binomial** | | | | **Gaussian GLM** | | | |
| --- | --- | --- | --- | --- | --- | --- | --- | --- |
| **Coefficients:** | **Est** | **Std. Err** | **t** | **p** | **Est** | **Std. Err** | **t** | **p** |
| (Intercept) | 5.43 | 0.04 | 136.2 | <0.01 | 228.1 | 11.97 | 19.1 | <0.01 |
| dvoBallymena | 0.03 | 0.07 | 0.46 | 0.65 | 4.32 | 21.24 | 0.20 | 0.84 |
| dvoColeraine | -0.03 | 0.05 | -0.64 | 0.52 | -7.16 | 14.54 | -0.49 | 0.62 |
| dvoDungannon | 0.07 | 0.05 | 1.33 | 0.19 | 16.91 | 16.37 | 1.03 | 0.30 |
| dvoEnniskillen | -0.19 | 0.05 | -3.76 | <0.01 | -40.68 | 15.56 | -2.62 | 0.01 |
| dvoLarne | 0.04 | 0.06 | 0.60 | 0.55 | 8.73 | 19.45 | 0.45 | 0.65 |
| dvoLondonderry | -0.04 | 0.07 | -0.49 | 0.62 | -10.37 | 22.39 | -0.46 | 0.64 |
| dvoNewry | 0.15 | 0.04 | 3.60 | <0.01 | 37.38 | 12.70 | 2.94 | 0.00 |
| dvoNewtownards | 0.01 | 0.04 | 0.21 | 0.84 | 1.96 | 12.94 | 0.15 | 0.88 |
| dvoOmagh | -0.07 | 0.05 | -1.58 | 0.11 | -15.72 | 13.55 | -1.16 | 0.25 |
| log(main_sett) | -0.18 | 0.13 | -1.35 | 0.18 | -41.28 | 39.38 | -1.05 | 0.29 |
| dvoBallymena:log(main_sett) | 0.43 | 0.15 | 2.82 | <0.01 | 91.43 | 46.07 | 1.98 | 0.05 |
| dvoColeraine:log(main_sett) | 0.23 | 0.14 | 1.69 | 0.09 | 52.99 | 41.41 | 1.28 | 0.20 |
| dvoDungannon:log(main_sett) | 0.20 | 0.15 | 1.34 | 0.18 | 47.73 | 45.98 | 1.04 | 0.30 |
| dvoEnniskillen:log(main_sett) | 0.13 | 0.14 | 0.92 | 0.36 | 31.30 | 41.39 | 0.76 | 0.45 |
| dvoLarne:log(main_sett) | 0.26 | 0.15 | 1.70 | 0.09 | 59.20 | 45.32 | 1.31 | 0.19 |
| dvoLondonderry:log(main_sett) | 0.43 | 0.16 | 2.61 | 0.01 | 87.94 | 49.30 | 1.78 | 0.07 |
| dvoNewry:log(main_sett) | 0.12 | 0.14 | 0.85 | 0.40 | 25.93 | 42.43 | 0.61 | 0.54 |
| dvoNewtownards:log(main_sett) | 0.12 | 0.14 | 0.87 | 0.39 | 28.93 | 42.80 | 0.68 | 0.50 |
| dvoOmagh:log(main_sett) | 0.16 | 0.14 | 1.12 | 0.26 | 38.31 | 43.46 | 0.88 | 0.38 |
