# Supplementary material for: Bovine tuberculosis breakdown duration in cattle herds: an investigation of herd, host, pathogen and wildlife risk factors
Source: PeerJ. 2020 Feb 3;8:e8319. doi: 10.7717/peerj.8319 (PMC7003687; doi:10.7717/peerj.8319)
Supplement: Table S6 — In other DVOs, increases in main sett density is associated with decreasing breakdown duration (difference = negative). These numbers are extracted from Supplementary Table 4 (univariable model) and Supplementary Table 5 (full model). The data from the Gaussian GLM (as opposed to the negative binomial GLM) are presented for illustrative purposes and for ease of interpretation (untransformed model coefficients). [file peerj-08-8319-s007.docx]

**Supplementary Material, Table 6**: The impact of main sett density on breakdown duration on a per-DVO basis, showing that in some DVOs, increases in main sett density is associated with increasing breakdown duration (Difference = positive). In other DVOs, increases in main sett density is associated with decreasing breakdown duration (difference = negative). These numbers are extracted from Supplementary Table 4 (univariable model) and Supplementary Table 5 (full model). The data from the Gaussian GLM (as opposed to the negative binomial GLM) are presented for illustrative purposes and for ease of interpretation (untransformed model coefficients).

|  | **Data from Table 5** | | | | | | **Data from table 4** |  |  |
| --- | --- | --- | --- | --- | --- | --- | --- | --- | --- |
|  | **Mean Breakdown Duration: full model, Fixed Effects estimate** | **Mean Breakdown Duration: full model, fixed effects estimate + intercept (228.07)** | **Fixed effects: full model, main sett estimate (GLM)** | **Mean Breakdown Duration: full model, fixed effects estimate + intercept + main sett** | **Interaction effects; dvo:main_sett** | ***Mean Breakdown Duration: full model, fixed effects estimate + intercept + main sett + interaction effects*** | ***Mean Breakdown Duration: Fixed Effects estimate*** | **Difference between breakdown length estimate (no interaction) & breakdown length estimate (interaction)** | **Difference** |
| Armagh (ref) | 228.07 | 228.07 | -41.28 | 186.79 | 186.79 | *186.79* | *239.75* | -52.96 | NEGATIVE |
| dvoBallymena | 4.32 | 232.39 | -41.28 | 191.11 | 91.43 | *282.54* | *199.55* | 82.99 | POSITIVE |
| dvoColeraine | -7.16 | 220.91 | -41.28 | 179.63 | 52.99 | *232.61* | *215.65* | 16.96 | POSITIVE |
| dvoDungannon | 16.91 | 244.98 | -41.28 | 203.70 | 47.73 | *251.43* | *243.12* | 8.31 | POSITIVE |
| *dvoEnniskillen* | -40.68 | 187.39 | -41.28 | 146.11 | 31.30 | *177.40* | *195.69* | -18.29 | NEGATIVE |
| dvoLarne | 8.73 | 236.80 | -41.28 | 195.52 | 59.20 | *254.72* | *227.15* | 27.57 | POSITIVE |
| dvoLondonderry | -10.37 | 217.70 | -41.28 | 176.42 | 87.94 | *264.36* | *193.06* | 71.31 | POSITIVE |
| *dvoNewry* | 37.38 | 265.45 | -41.28 | 224.17 | 25.93 | *250.10* | *267.15* | -17.06 | NEGATIVE |
| *dvoNewtownards* | 1.96 | 230.03 | -41.28 | 188.75 | 28.93 | *217.67* | *228.08* | -10.41 | NEGATIVE |
| *dvoOmagh* | -15.72 | 212.35 | -41.28 | 171.07 | 38.31 | *209.38* | *213.62* | -4.24 | NEGATIVE |
