# Supplementary material for: Bovine tuberculosis breakdown duration in cattle herds: an investigation of herd, host, pathogen and wildlife risk factors
Source: PeerJ. 2020 Feb 3;8:e8319. doi: 10.7717/peerj.8319 (PMC7003687; doi:10.7717/peerj.8319)
Supplement: Table S7 [file peerj-08-8319-s008.docx]

**Supplementary Material, Table 7**: Results of the negative binomial count model of breakdown duration, with DVO omitted from the random effects and *log* main sett included as a fixed effect, instead of *log* patch prevalence (untransformed model coefficients).

| Random Effects | **Variance** | **Std.Dev.** |  |  |
| --- | --- | --- | --- | --- |
| herd_id (Intercept) | 0.048 | 0.229 |  |  |
| year (Intercept) | 0.007 | 0.076 |  |  |
|  |  |  |  |  |
|  |  |  |  |  |
|  |  |  |  |  |
| Fixed effects | **Estimate** | **Std. Error** | **z value** | **P** |
| (Intercept) | 5.030 | 0.038 | 132.146 | <0.001 |
| log(herd_size) | 0.045 | 0.004 | 10.121 | <0.001 |
| log(outbreak_reactors) | 0.056 | 0.006 | 8.774 | <0.001 |
| log(main_sett) | 0.081 | 0.012 | 6.666 | <0.001 |
| log(MLVA_Richness) | 0.521 | 0.014 | 36.679 | <0.001 |
| LRS_binary1 | 0.134 | 0.011 | 11.626 | <0.001 |
| associated_herds_binary1 | 0.096 | 0.012 | 8.176 | <0.001 |
| previous_breakdown | 0.021 | 0.013 | 1.696 | 0.090 |
