# Supplementary material for: Bovine tuberculosis breakdown duration in cattle herds: an investigation of herd, host, pathogen and wildlife risk factors
Source: PeerJ. 2020 Feb 3;8:e8319. doi: 10.7717/peerj.8319 (PMC7003687; doi:10.7717/peerj.8319)
Supplement: Table S8 [file peerj-08-8319-s009.docx]

**Supplementary Material, Table 8**: Results of the negative binomial count model of breakdown duration, with DVO omitted from the random effects and *log* patch prevalence included in the fixed effects*. Log* main sett is included as a fixed effect (untransformed model coefficients).

| Random Effects | **Variance** | **Std.Dev.** |  |  |
| --- | --- | --- | --- | --- |
| herd_id (Intercept) | 0.047 | 0.231 |  |  |
| year (Intercept) | 0.007 | 0.084 |  |  |
|  |  |  |  |  |
|  |  |  |  |  |
|  |  |  |  |  |
| Fixed effects | **Estimate** | **Std. Error** | **z value** | **p** |
| (Intercept) | 5.002 | 0.047 | 105.459 | <0.001 |
| log(herd_size) | 0.045 | 0.004 | 10.099 | <0.001 |
| log(outbreak_reactors) | 0.056 | 0.006 | 8.707 | <0.001 |
| log(mean_patch_prev) | 0.012 | 0.012 | 1.009 | 0.313 |
| log(main_sett) | 0.078 | 0.013 | 6.189 | <0.001 |
| log(MLVA_Richness) | 0.521 | 0.014 | 36.690 | <0.001 |
| LRS_binary1 | 0.135 | 0.012 | 11.670 | <0.001 |
| associated_herds_binary1 | 0.096 | 0.012 | 8.196 | <0.001 |
| previous_breakdown | 0.020 | 0.013 | 1.568 | 0.117 |
